# Epidemiological monitoring and genetic variation analysis of pathogens associated with porcine viral diarrhea in southern China from 2021 to 2023

**DOI:** 10.3389/fmicb.2024.1303915

**Published:** 2024-03-20

**Authors:** Fanfan Zhang, Yangyang Luo, Cui Lin, Meifang Tan, Peiwei Wan, Baobao Xie, Ligen Xiong, Huayuan Ji

**Affiliations:** ^1^Institute of Animal Husbandry and Veterinary Medicine, Jiangxi Academy of Agricultural Sciences, Nanchang, Jiangxi, China; ^2^Wen’s Foodstuff Group Co., Ltd., Wen’s Group Research Institute, Yunfu, Guangdong, China; ^3^Jiangxi Biological Vocational College, Nanchang, Jiangxi, China; ^4^Dabeinong Technology Co., Ltd. of Jiangxi, Nanchang, Jiangxi, China

**Keywords:** porcine diarrhea, prevalence, porcine epidemic diarrhea virus, porcine deltacoronavirus, swine acute diarrhea syndrome coronavirus, porcine rotavirus

## Abstract

Large-scale outbreaks of virus-associated severe diarrhea have occurred in pig populations since 2010. To investigate the prevalence and genetic evolution of the diarrhea-associated viruses responsible for the outbreaks, we tested 1,791 diarrhea samples collected from 213 pig farms in five provinces in southern China between 2021 and 2023. The test results showed that porcine epidemic diarrhea virus (PEDV) was the most frequently detected virus. The prevalence rates ranged from 47.40 to 52.22% in samples and 76.06% (162/213) in pig farms. Porcine rotavirus (PoRV) was the second common virus, with prevalence rates ranging from 25.81 to 50.81% in samples and 72.77%(155/213) in pig farms. Porcine delta coronavirus (PDCoV) was the third common virus, with prevalence rates ranging from 16.33 to 17.48% in samples and 38.50% (82/213) in pig farms. The detection rates of both transmissible gastroenteritis virus (TGEV) and porcine acute diarrheal syndrome coronavirus (SADS-CoV) were very low, less than 1.01% in samples and less than 3.76% in pig farms. In this study, we found SADS-CoV only in piglet diarrhea samples from Jiangxi, Guangdong, and Guangxi provinces in China, with a prevalence rate of 5.16% (11/213) in pig farms. Co-infection with these diarrhea-associated viruses is a common occurrence. The most common co-infections were PEDV and PoRV, with a prevalence rate of 6.64% (119/1,791), followed by PDCoV and PoRV, with a prevalence rate of 4.19% (75/1,791). Phylogenetic analyses showed that PEDV and PEDV variants prevalent in southern China during the past three years clustered into genotype GIIb and recombinant PEDV subtypes. Among the currently endemic PEDV, the most common mutations occurred in the collagenase equivalent (COE) and epitope regions of the spike gene. PoRV strains were mainly dominated by the G9 subtype, followed by the G5, G3 and G4 subtypes. Our results suggest that variant PEDV, PDCoV and PoRV are the main pathogens of swine diarrhea, and singular- or co-infection with pathogenic enteric CoV is common in pig herds in southern China. Therefore, prevention and control of porcine viral diarrhea should be given high attention.

## 1 Introduction

Viral diarrhea is an important disease that seriously damages the pig industry, with porcine epidemic diarrhea virus (PEDV), porcine delta coronavirus (PDCoV), porcine transmissible gastroenteritis virus (TGEV), and porcine rotavirus (PoRV) being the four main pathogens (Liu and Wang, 2021; Zhang et al., 2022). PEDV can infect pigs of all ages, causing watery diarrhea and vomiting accompanied by anorexia and depression. Morbidity approaches 100% in piglets, but can vary in sows. The virus was first reported by British researchers in 1971, and the disease was first reported in China in 1980, and is currently circulating in all major pig producing countries in the world (Jang et al., 2023). PDCoV is a newly discovered porcine enteropathogenic coronavirus in 2014, which can cause vomiting, diarrhea, dehydration and even death in pigs of all ages, especially piglets, with clinical symptoms similar to those of PED (Zhang F. et al., 2017). TGEV was first isolated in the United States in 1946 and can cause acute gastrointestinal infections in pigs of all ages. Clinical symptoms are extremely similar to PDCoV, with mortality rates of up to 100 per cent in piglets and very few deaths in adult pigs (Zhang et al., 2019). Porcine rotavirus disease is an acute intestinal infectious disease caused by PoRV. In 1974, Woode and Bridge first isolated rotavirus from pigs in the UK and the clinical signs are similar to those of PEDV. Pigs of different ages can be infected, but clinically the most obvious symptoms are seen in piglets within 2 months of age and the mortality rate is as high as 95%. With increasing age, resistance gradually increases and medium and large pigs have strong resistance and clinically it is mostly a latent infection process and infection of pregnant pigs can be transmitted to piglets through the placenta (Ryu et al., 2021). Epidemics caused by PDCoV, PEDV, TGEV, and PoRV not only have similar clinical symptoms, but also often have co-infections and secondary infections that increase herd morbidity and mortality, making these pathogens important pathogens that are currently harming the pig industry (Hou et al., 2023). SADS-CoV was first detected in Guangdong in February 2017, causing a relatively large outbreak of piglet diarrhea with clinical symptoms similar to those of PED, and is a novel porcine coronavirus discovered before the emergence of the COVID-19 outbreak (Zhou et al., 2018). To date, SADS-CoV has only been a regional sporadic infection, and has only been found in a few pig farms in Guangdong, Fujian and Guangxi provinces. However, SADS-CoV has the potential to be broadly species-specific, especially infecting a wide range of human respiratory and intestinal progenitor cells, which should be highly regarded as a potential public health hazard, and the possibility of cross-species transmission of SADS-CoV to other species or to human beings in the future. Strengthening the epidemiological investigation of the above five pathogens is an important basis for good prevention and control of viral diarrhea in pigs.

In this study, we investigated five major porcine diarrhea-associated viruses including PEDV, PDCoV, TGEV, PoRV, and SADS-CoV in five provinces (Guangdong, Guangxi, Jiangxi, Fujian and Hunan) from porcine diarrhea samples collected from 2021 to 2023. Sequencing analysis of the PEDV S1 gene and PoRV VP7 was performed to elucidate the genetic characteristics of PEDV and PoRV in the southern provinces of China.

## 2 Materials and methods

### 2.1 Animal ethics statement

All samples were collected on commercial pig farms by pig veterinarians during routine diagnostic sampling after permission from the farm owner. No specific permits from an animal ethics committee were required.

### 2.2 Sample collection and viral RNA/DNA extraction

From 2021 to 2023, a total of 1,791 diarrheal samples from 213 pig farms in five provinces (Guangdong, Guangxi, Fujian, Jiangxi and Hunan) in southern China were submitted to the Key Laboratory for Animal Health at the Department of Preventive Veterinary Medicine in Jiangxi Agricultural University and Research Institute of Guangdong Wen’s Food Group Co., Ltd. for diagnosis. These samples included small intestinal contents and tissues (*N* = 774), feces (*N* = 1,017) from domestic pigs (*Sus scrofa*) of different age groups with diarrhea: 223 samples from sows, 1,192 samples from suckling piglets, 219 samples from nursery pigs and 157 samples from finishing/adult pigs (Table 1). The small intestinal contents and tissues samples used in this study were obtained from the dead piglets and the fecal samples were non-invasively collected immediately after excretion from diarrheal pigs from premises by veterinarians in these farms and then submitted to laboratory. All samples were recorded and aliquoted into 5 mL eppendorf tubes and stored at −80°C until tested.

**Table 1:** Categorization of detection results on porcine diarrhea associated viruses of samples collected from 2021 to 2023.

Classifications	Sample No.	Viruses [Positive rate % (Number)]				
		PEDV	PRoV	PDCoV	TGEV	SADS-CoV
Year						
2021	496	52.22 (259)	25.81 (128)	16.33 (81)	1.01 (5)	2.62 (13)
2022	738	49.73 (367)	34.01 (251)	17.48 (129)	0.95 (7)	3.66 (27)
2023	557	47.40 (264)	50.81 (283)	17.41 (97)	0.54 (3)	0.00 (0)
Total	1,791	49.69 (890)	36.96 (662)	17.14 (307)	0.84 (15)	2.23 (40)
Province						
Guangdong	793	47.54 (377)	36.44 (289)	15.89 (126)	0.88 (7)	1.01 (8)
Guangxi	501	50.70 (254)	3,713 (186)	18.16 (91)	1.20 (6)	4.19 (21)
Jiangxi	372	53.49 (199)	38.98 (145)	19.35 (72)	0.54 (2)	2.96 (11)
Fujian	82	47.56 (39)	30.49 (25)	17.07 (14)	0.00 (0)	0.00 (0)
Hunan	43	48.84 (21)	39.53 (17)	9.30 (4)	0.00 (0)	0.00 (0)
Sample type						
Intestine	774	58.79 (455)	36.30 (281)	25.58 (198)	0.00 (0)	3.75 (29)
Feces	1,017	42.77 (435)	37.46 (381)	10.72 (109)	1.47 (15)	1.08 (11)
Growing stage						
Sow	223	46.19 (103)	31.39 (70)	12.56 (28)	2.24 (5)	1.79 (4)
Suckling piglet	1,192	54.95 (655)	41.28 (492)	21.22 (253)	0.67 (8)	2.68 (32)
Nursery pig	219	38.81 (85)	29.22 (64)	6.85 (15)	0.46 (1)	0.91 (2)
Finishing pig	157	29.94 (47)	21.66 (34)	7.01 (11)	0.64 (1)	1.27 (2)

PEDV, Porcine epidemic diarrhea virus; PDCoV, Porcine deltacoronavirus; TGEV, Transmissible gastroenteritis virus; PoRV, Porcine rotavirus; SADS-CoV, Swine acute diarrhea syndrome coronavirus.

### 2.3 Detection of porcine diarrhea associated viruses

Total viral DNA/RNA was extracted using TaKaRa MiniBEST Viral RNA/DNA Extraction Kit Ver.5.0 (Takara, Dalian, China) following the manufacturer’s instruction, and reverse transcribed into first-strand cDNA using HiScript II 1st Strand cDNA Synthesis Kit (Vazyme, Nanjing, China) by random hexamer primers. The steps of reverse transcription included denaturation of the RNA template and synthesis of the 1st strand cDNAs. A total volume of 20 μL of reaction solution was instantly centrifuged and placed in an applied biosystems PCR cycler (Applied Biosystems, Waltham, MA USA). The RNA template is converted to complementary (c)DNA and used as a template for triplex real-time PCR or commercial singleplex real-time PCR. The previously established PCR protocols were used to test four major diarrhea-associated viruses, PEDV, PDCoV, TGEV, and PoRV. The newly emerged SADS-CoV was tested by a method previously established in our laboratory (primer sequence information is presented in the Supplementary Table S2) (Zhang et al., 2019). Data were analyzed based on the discriminations of year, pig growing stage, and sampling area.

### 2.4 Analysis of PEDV S1 gene and PoRV VP7 gene

To elucidate the molecular characteristics of the S1 gene of PEDV and the VP7 gene of PoRV, representative PEDV- and PoRV-positive samples were amplified, cloned and then sequenced on the basis of previous studies. The sequence fragments of the PCR products were assembled and annotated using SeqMan software (DNASTAR, Madison, USA). Nt and aa sequences of the PEDV S1 and PoRV VP7 genes were aligned by using the Clustal W Method in DNAStar software (Version 7.10). Phylogenetic trees were generated based on the PEDV S1 and PoRV VP7 genes by using the neighbor-joining method of Molecular Evolutionary Genetics Analysis (MEGA version 7.0) with a bootstrap value of 1,000 replicate datasets. The obtained strains and 29 reference strains from the GenBank database were used for this purpose.

### 2.5 Statistical analysis

Statistical analyses were performed using SPSS software V20.0 (IBM Corporation, Chicago, USA). A *p*-value of < 0.05 was set as the statistically significant level.

## 3 Results

### 3.1 Prevalence of PEDV, PoRV, PDCoV, TGEV, and SADS-CoV

In this study, 1,791 samples from 213 pig farms in five southern provinces of China were detected from 2021 to 2023. The results showed that variant PEDV was the main virus associated with severe diarrhea, with prevalence rates ranging between 47.40 and 52.22%. With good biosecurity measures and widespread use of the PEDV variant vaccine, the PEDV detection rate shows a downward trend year by year during the period 2021–2023 (Table 1). In addition to PEDV, PoRV, PDCoV, TGEV, and SADS-CoV have also been observed among some diarrhea samples tested. Interestingly, the positive rate of PoRV has been increasing in recent years, surpassing PDCoV as the second pathogen causing piglet diarrhea, with a positive rate up to 50.81%. In 2023, the detection rate of PoRV has surpassed that of PEDV, and it has become the most important killer of piglet diarrhea. SADS-CoV was found only in a small number of pig farm samples collected in Guangdong, Guangxi and Jiangxi provinces in this study, but the SADS-CoV was not observed in samples collected in Fujian and Hunan provinces. The SADS-CoV positivity rate of diarrheic piglets in Guangdong, Guangxi and Jiangxi provinces was 1.01% (8/793), 4.19% (21/501) and 2.96% (11/372), respectively. The detection rate of TGEV was low (<1.5%).

In the context of the sample source, the small intestines of suckling piglets showed the highest detection rate of PEDV (63.31%), followed by feces (52.72%). Likewise, the detection rates of PoRV from small intestine and feces were 29.66 and 7.92%, respectively (Table 1). As to the growing stage of pigs, PEDV was frequently detectable in pigs of all ages, followed by PoRV. PEDV infection was more common in sows (46.19%) and suckling piglets (54.95%), and similar results were observed for PoRV.

### 3.2 Co-infections of diarrhea-associated viruses in pigs in southern China

In this study, Mono- and co-infection frequency was analyzed. Among the 1,791 clinical samples, the rates of mono-infection of PEDV, PDCoV, TGEV, SADS-CoV and PoRV in samples tested were 38.86% (696/1,791), 8.77% (157/1,791), 0.22% (4/1,791), 1.45% (26/1,791), and 24.23% (434/1,791), respectively (Table 2). The prevalence rate of co-infections due to two or more diarrhea-associated pathogens ranged from 0.11 to 9.87% in 2021–2023. The most common co-infection was PEDV with PoRV, with a mean positivity rate of 6.64% (119/1,791), with the highest positivity rate of 9.87% (55/557) in 2023. The mean co-infection rates of PEDV with PDCoV, PEDV with TGEV, PEDV with SADS-CoV, PDCoV with PoRV, TGEV with PoRV, and SADS-CoV with PoRV had mean dual infection rates of 2.29, 0.28, 0.22, 4.19, 0.22 and 0.39%, respectively. It should be noted that the co-infection rate of PEDV and PoRV is increasing year by year, with a positive rate of 4.64, 5.56, and 9.87% in 2021–2023, respectively. Meanwhile, triple infections were found in clinical samples, with PEDV, PDCoV, and PoRV infections being the most common, with an average positivity rate of 1.90%. In addition, two cases were found to be co-infected with PEDV, TGEV and PoRV, and three cases were co-infected with PEDV, SADS-CoV, and PoRV.

**Table 2:** Mono- and co-infections of PEDV, PDCoV, TGEV, PoRV, and SADS-CoV in samples from southern China during 2021 to 2023.

Type of infection	2021		2022		2023		Total	
	Positive	Positive rate, %	Positive	Positive rate,%	Positive	Positive rate (%)	Positive	Positive rate, %
PEDV only	217	43.75	295	39.97	184	33.03	696	38.86
PDCoV only	43	8.67	68	9.21	46	8.26	157	8.77
TGEV only	2	0.40	1	0.14	1	0.18	4	0.22
SADS-CoV only	9	1.81	17	2.30	0	0.00	26	1.45
PRoV only	79	15.93	166	22.49	189	33.93	434	24.23
PEDV+PDCoV	12	2.42	17	2.30	12	2.15	41	2.29
PEDV+TGEV	1	0.20	2	0.27	2	0.36	5	0.28
PEDV+SADS-CoV	1	0.20	3	0.41	0	0.00	4	0.22
PEDV+PRoV	23	4.64	41	5.56	55	9.87	119	6.64
PDCoV+PRoV	19	3.83	30	4.07	26	4.67	75	4.19
TGEV+PRoV	1	0.20	3	0.41	0	0.00	4	0.22
SADS-CoV+PRoV	2	0.40	5	0.68	0	0.00	7	0.39
PEDV+PDCoV+PRoV	7	1.41	14	1.90	13	2.33	34	1.90
PEDV+TGEV+PRoV	1	0.20	1	0.14	0	0.00	2	0.11
PEDV+SADS-CoV+PRoV	1	0.20	2	0.27	0	0.00	3	0.17

### 3.3 Molecular characterization and phylogeny of PEDVs circulating in southern China during 2021 to 2023

To elucidate the genetic characteristics of PEDVs circulating in southern China during 2021 to 2023, the S genes of 9 representative strains of PEDV were sequenced, and analyzed. Phylogenetically, the S regions (aa 1 ~ 794) of the 9 strains of PEDV identified in this study and other 34 selected reference PEDV strains were divided into two genotypes (genotype I: GI and genotype II: GII). All nine PEDV strains identified in this study were classified in GII (subgroup GIIb and Recombinant PEDV) (Figure 3A). Eight epidemic wild strains were located on the GIIb branch, and one belonged to the group GII Recombinant PEDV type, which was distantly related to the vaccine (CV777, Attenuated DR13, JS2008, etc.) strains located in group G1b. The strains in the group GIIb belonged to the epidemic strains, which were more similar to the strains of the original outbreaks in China (AJ1102, FL2013, GD1, etc.). In addition, the eight PEDV strains isolated in this study were distantly related to MEX/104/2013 (Mexico), KNU-1305 (South Korea) and IA1 (USA). JX3 belongs to the Recombinant PEDV type of group GII, and is closer to the LS-23 strain and the CH/HB/ZJK02 strain.

**Figure 1:**
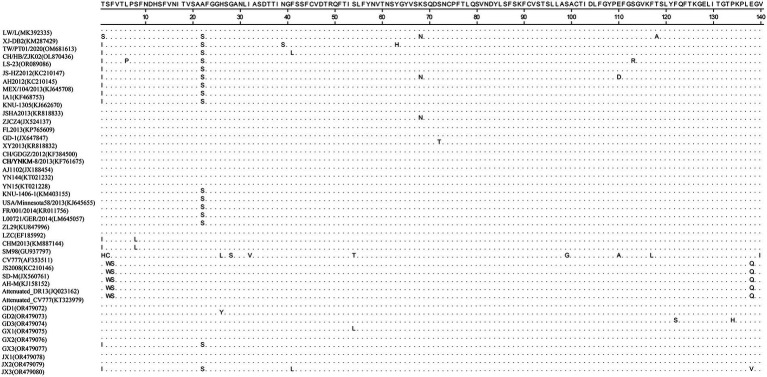
Amino acid alignment results based on the S protein sequences of PEDVs. The neutralizing epitopes COE are shown.

**Figure 2:**
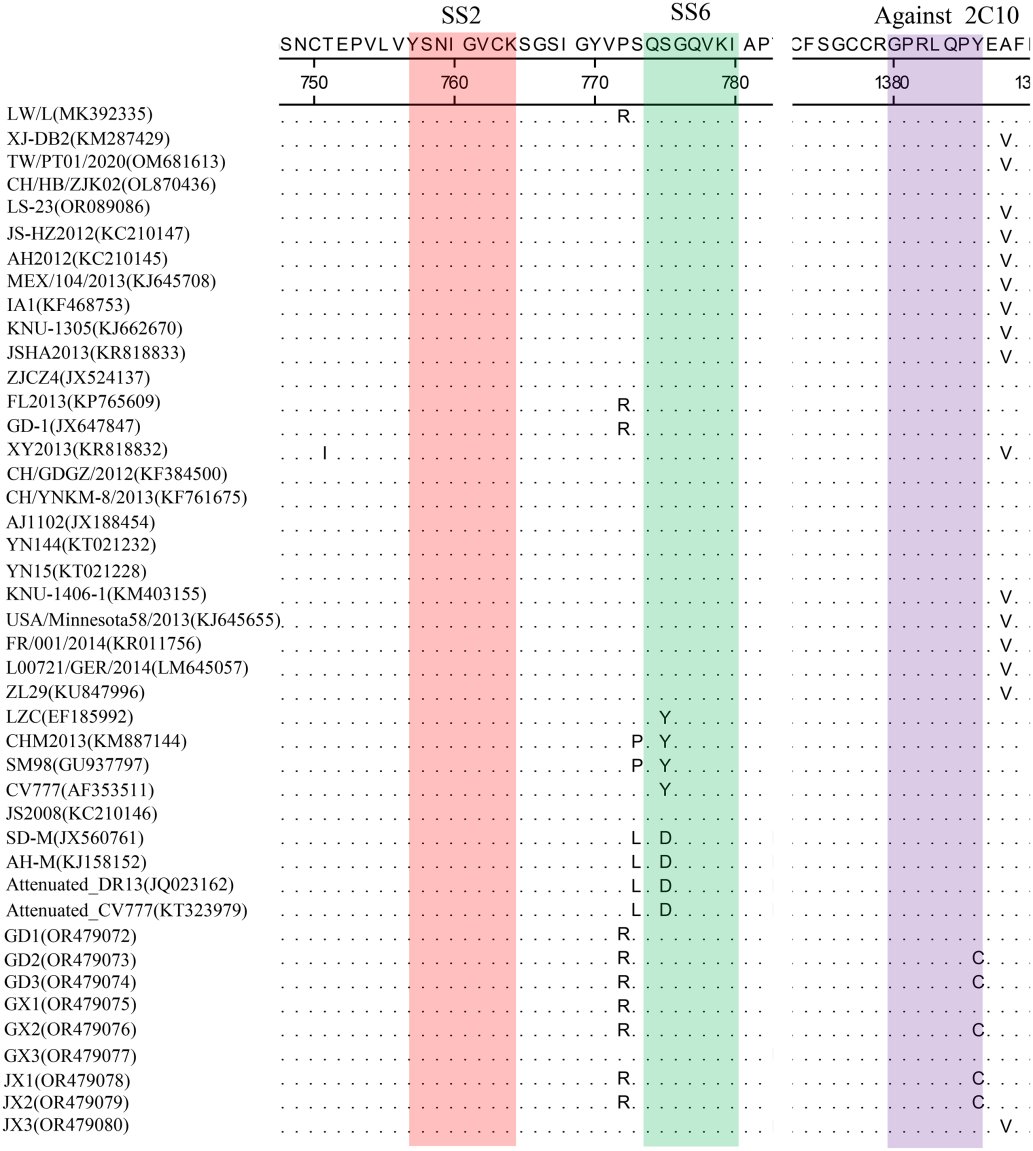
Amino acid alignment results based on the S protein sequences of PEDVs. The neutralizing epitopes SS2 (red), SS6 (green) and against 2C10 (purple) are shown.

Homology analyses were performed between the nine PEDV strains identified in this study and other 34 selected reference PEDV strains based on the S gene. The results showed that the nucleotide (nt) and amino acid (aa) homologies of all field strains were 98.6–100% and 98.4–99.9%, respectively, and the homologies with the GI strains were 92.9–95.6% and 91.7–95.4%, respectively, and the homologies with the GII strains were 96.9–99.8% and 96.0–99.6% for nt and aa, respectively (Supplementary Table S3). The S protein epitopes of the nine PEDV strains were analyzed, and SS2 and 2C10 were relatively conserved, with the major variants located in the COE region and SS6. In comparison with the classical strain CV777, this isolate had up to 15 amino acid mutations in the S protein and in the COE segment of the antigenic epitope, including H500I/T, C501S, A521S, L525H/Y, S527G, V531I, F540L, T543S/L, G598S, A609E, L616F, F621F, P633H, E637V, and I639V, but no amino acid insertions or deletions were found (Figure 1). Point mutations in SS6 included Y775S (Figure 2).

### 3.4 Molecular characterization and phylogeny of PoRVs circulating in southern China during 2021 to 2023

**Figure 3:**
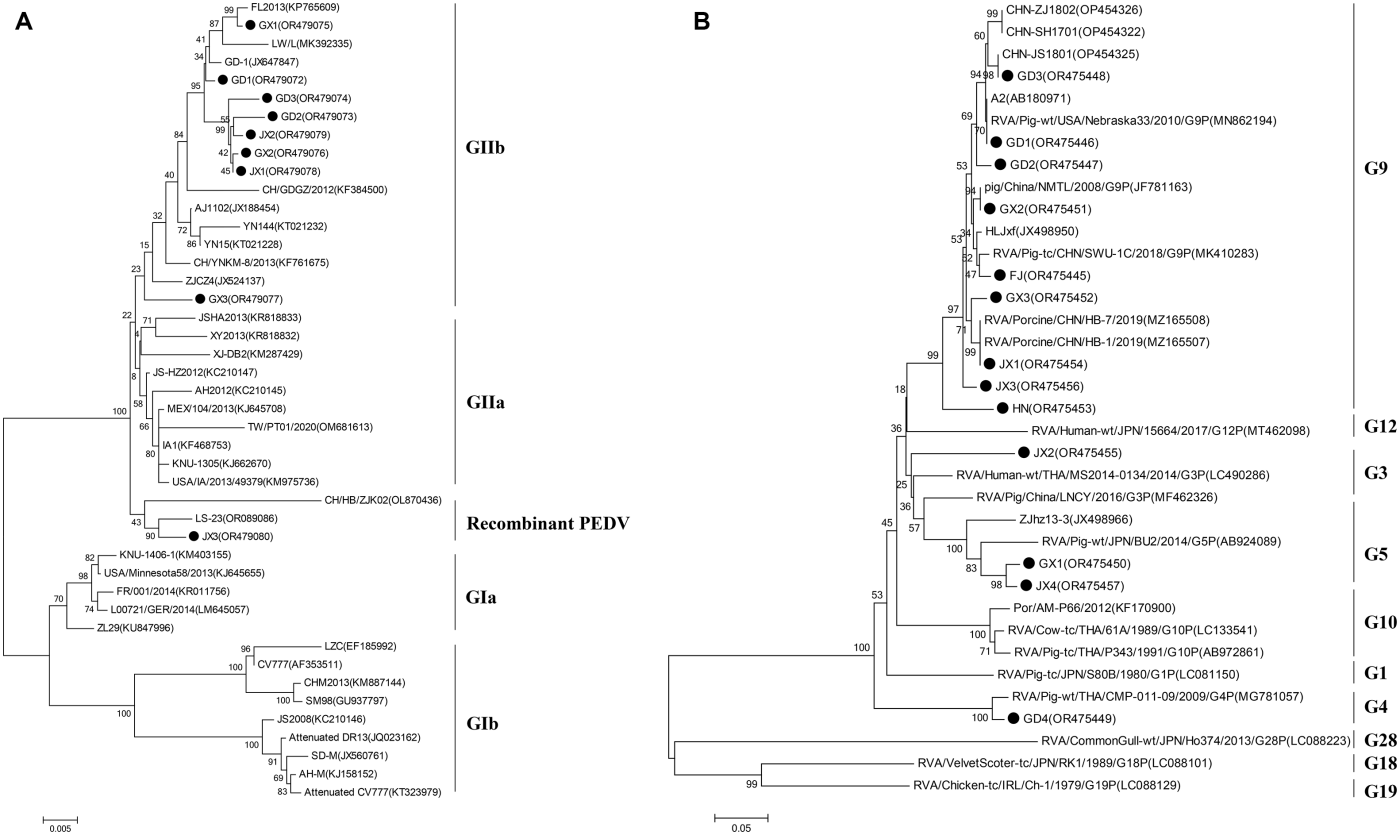
Phylogenetic analysis on the animo acid sequences of the S protein (full length sequence) of selected PEDV **(A)** and the VP7 protein (full length sequence) of selected PoRV **(B)** strains from different countries. Solid black circle indicates the strains determined in this study. The tree was constructed using the neighbor-joining method (bootstrap resampling = 1,000 replications) in the MEGA software package, version 7.0.

VP7 gene sequences of PoRV strains (*N* = 13) were obtained from representative PoRV-positive samples. To analyze the molecular characteristics and phylogenetic relationships among different PoRV isolates, the 13 PoRV strains determined in this study and the 23 PoRV reference strains retrieved from GenBank were used. The nt identities among the 13 PoRV isolates determined in this study ranged from 95.6 to 98.2% and the aa identities ranged from 76.6 to 97.9%; the nt identities with the reference strains from China and the rest of the world ranged from 57.6 to 100% and the aa identities ranged from 50.9 to 100% (Supplementary Table S4). The genetic evolution tree showed that there were four genotypes of the VP7 gene in the 13 sample sequences, namely, one strain of G3 genotype, one strain of G4 subtype, two strains of G5 subtype, and nine strains of G9 genotype. In the evolutionary tree, we can see a separate branch of the G3-subtype sequence for the JX2 strain, which is close to the RVA/Human-wt/THA/MS2014-0134/2014/G3P strain; and GD1, GD2, GD3, GX2, FJ, GX3, JX1, JX3, and HN all belonged to the G9 subtype (Figure 3B).

## 4 Discussion

Porcine diarrheal disease is a major problem plaguing the pig industry, and among the many causes of porcine diarrhea, porcine viral diarrheal disease has always been the main influencing factor. Common viruses that cause swine diarrhea include PEDV, TGEV, PDCoV, SADS-CoV, and PoRV (Wang et al., 2019; Zhang et al., 2019; Jung et al., 2020). Infection of pigs with these viruses results in diarrhea that affects energy intake and growth and development of individual pigs, and in the case of piglets the fate of the disease is likely to be death. Relevant studies have shown that PEDV dominates the pathogens of porcine viral diarrhea in China (Lee, 2015; Wang et al., 2016, 2019).

In order to understand the main pathogens causing diarrhea in pigs in southern China and the degree of infection, a total of 1,791 clinical diarrhea samples from 213 pig farms in five provinces in southern China were collected from January 2021 to August 2023 for nucleic acid testing of the five pathogens mentioned above, and the results were subjected to data counting and analysis, and the results showed that PEDV infection was the most serious in diarrheic pig herds. The positive detection rate of the samples was 49.67%. These results were consistent with other studies conducted in other parts of China during the same period (Li et al., 2022). Zhang et al. investigated samples from 29 provinces in China between 2011 and 2014 and found that the detection rate of PEDV in diarrheal pigs ranged from 61.10 to 78.49%(Zhang et al., 2019). A study in 2016–2017 that included 116 diarrheal samples from six provinces in China showed that the PEDV prevalence rate was 52.60 per cent (Zhang Q. et al., 2017). Jia et al. detected 672 diarrhea samples collected in Northeast China from 2017 to 2018, and the results showed 19.05% (128/672), 4.32% (29/672), and 3.87% (26/672) positive rates for PEDV, PoRV, and PDCoV, respectively (Jia et al., 2019)。These data suggest that PEDV infections in pigs are common in China, the largest pork-producing country. However, according to the literature, before 2018, the viral diarrhea pathogen in Chinese pigs was mainly dominated by PEDV, and most of the PoRV infections in China were sporadic or local, with no large-scale outbreaks. According to the results of this survey, PEDV infections showed an overall decreasing trend, which may be closely related to the decline in pig inventory and the general strengthening of biosecurity management in pig farms after the occurrence of the African swine fever outbreak in the country in 2018. From 2021 to 2023, the detection rate of PoRV positivity increased each year. In 2023, the detection rate of PoRV will exceed that of PEDV for the first time, making it the most important pathogen causing viral diarrhea in pigs. At present, the prevalent serotypes of porcine rotavirus have changed significantly, evolving from the original G5 type to G9 type, with G9 type accounting for more than 80% of the total, while there are multiple serotypes such as G5 and G4. The serotype of the existing porcine rotavirus vaccine in the triple live vaccine for porcine infectious gastroenteritis, porcine epidemic diarrhea and porcine rotavirus is still G5, and there is almost no cross-protection between different serotypes of rotavirus, which is not able to produce an effective preventive effect for the current porcine rotavirus epidemic strains.

PDCoV was first detected in Hong Kong, China, in 2012 and broke out in the United States in 2014 (Song et al., 2015). In our study, we found that PDCoV was the third most common prevalent virus, which is similar to our previous findings. Ding et al. reported that the prevalence rate of PDCoV was as high as 36.18% in faucal samples from nine provinces in China between 2015 and 2017 (Ding et al., 2020). Therefore, PDCoV is also common in pigs in China.

TGEV used to be an important pathogen associated with diarrhea in most pig-producing countries/regions of the world (Li et al., 2022; Sun et al., 2023). However, the clinical cases and impact of TGEV have been limited in recent decades. In this study, we found that TGEV was only sporadically detected during 2021–2023, which is consistent with previous reports (Zhang et al., 2019). SADS-CoV, an emerging coronavirus causing acute diarrhea in suckling piglets, was first detected in Guangdong Province in southern China in 2017 (Zhou et al., 2018). It was subsequently reported in Fujian and Guangxi. In this study, SADS-CoV was detected in some samples from Jiangxi, Guangdong and Guangxi provinces, and the positive rate of the virus was 2.23% (40/1,791). SADS-CoV infections showed an upward trend, and comprehensive prevention and control should be strengthened to avoid the emergence of a large-scale epidemic of SADS-CoV. Sows (46.19%) and lactating piglets (54.95%) were infected with PEDV at a higher rate, as were PDCoV and PoRV infections. These data suggest that the degree of diarrhea may be related to differences in pig resistance, with adult pigs being more likely to be cryptically infected, and lactating piglets having the highest morbidity and mortality rates and being the most damaging. Co-infections with diarrhea-associated coronaviruses are common. Thus, the diagnosis of porcine diarrhea has become increasingly complex, and accurate differential diagnosis can only be made by laboratory tests because diarrhea caused by different enteric pathogens has similar clinical features.

In this study, we conducted a statistical analysis of the five viruses’ single and mixed infections, and found that the viral infections in diarrheal pig herds in southern China were dominated by single infections, mainly PEDV and PoRV single infections, with the infection rates of 38.86 and 24.23%, respectively; among the mixed infections, PDEV+PoRV was the most common, with the positivity rate of 2.29%, which was basically in line with the results of other studies (Wang et al., 2016; Zhang et al., 2019; Jung et al., 2020). In this study, it was found that most of the farms co-infected with PEDV and other pathogens (e.g., PoRV and PDCoV) had a history of epidemics of other pathogens (e.g., PoRV or PDCoV) before the emergence of PEDV. It has been demonstrated that mixed infections of PEDV+TGEV increase PEDV replication and lead to higher disease and death rates of piglets, but mixed infection of PEDV with other pathogens can promote virus replication or increase its pathogenicity needs further study (Sungsuwan et al., 2020). Overall, although PEDV is still the main pathogen causing diarrhea in piglets in different regions of China, the prevalence rate of other enteroviruses varies greatly in different regions, e.g., the detection rate of PEDV and PDCoV is higher than that of PoRV and TGEV in Xinjiang region, and the results of the present study showed that the detection rate of PEDV and PoRV is higher than that of PDCoV and TGEV, which may be due to the differences in geographic regions and pig farms’ management. Management differences.

The PEDV S gene is highly variable and is often used as a molecular marker for the analysis of viral genotypes and their variability (Cui et al., 2019). The homology between the S gene sequences of the nine PEDV strains detected in this study ranged from 98.6 to 100%, and the homology of amino acid sequences ranged from 98.4 to 99.9%; the S gene sequences of the nine strains showed the highest homology compared with the corresponding gene sequences of the reference strain of PEDV of type GII, which suggests that all of the above strains belong to the type GII. Further genetic evolutionary analysis based on the S gene sequences showed that eight strains of PEDV belonged to the GII-b subtype, and one strain was the Recombinant PEDV subtype. Most commercially available PEDV vaccines are of GI (e.g., strain CV777) and GII-a subtypes (e.g., strain AJ1102), and a large number of studies have demonstrated that the PEDV CV777 vaccine strain is unable to provide sufficient immunoprotection against the attack of endemic strains of PEDV of GII type. In contrast, vaccines based on GII-a subtype PEDV provide only partial immunological protection against attack by GII-b subtype PEDV, but do not fully guarantee protection against infection or disease (Liu et al., 2019; Zhang et al., 2020). The PEDV S protein is involved in fusion of the virus with host cell receptors and stimulation of the body’s production of neutralizing antibodies, etc. Based on the amino acid sequence characteristics of the S protein, it can be divided into the S1 and S2 proteins. The S1 protein contains three neutralizing epitopes: the collagenase equivalence (COE) region (aa499 ~ aa638), epitope SS2 (aa748 ~ aa755), and epitope SS6 (aa764 ~ aa771). Compared with the CV777 strain, the nine PEDV strains detected in this study had partial amino acid mutations in the above and epitope regions of the PEDV S amino acid sequence; furthermore, compared with the Gll-a subtype PEDV vaccine strain (AJ1102) developed in China, part of the PEDV (GD3) S1 protein, aa633, was mutated from P The amino acid of this site is located in the epitope of the COE region of the S1 protein, but whether its mutation (P → H) affects the epitope structure needs to be further explored.

The evolution of PEDV in the wild has rapidly accelerated over the last decade. Since the end of 2010, highly pathogenic strains of PEDV have appeared in China and have subsequently been detected in other countries (Chen et al., 2012; Pan et al., 2012; Sun et al., 2012). Although CV777-based PEDV vaccines have been widely used in China, high morbidity and mortality of neonatal piglets infected with mutated PEDV are still common. Genetic analyses based on circulating strains of PEDV showed that all PEDV can be classified into two genotypes, GI type and GII type: (1) GI type includes classical strains of PEDV, represented by CV777 and strains that appeared before 2010; and (2) GII type includes mutant strains found after 2010. Mutations such as insertions and deletions are frequently observed in the complete genome of variant PEDV, most of which are located in spiking genes, including the antigenic epitope region (Yu et al., 2023). The S-protein of PEDV is an important structural protein of coronaviruses, and is thought to encode antigenic determinants, particularly the viral neutralization epitope. Based on phylogenetic analyses of the S protein, all nine strains of viruses identified in this study belong to the GII variant of PEDV (Kristen-Burmann et al., 2023). Mutations in the neutralization epitopes on the S protein of PEDV have been reported in comparison to conventional PEDV strains, including the CO-26 K-equivalent epitopes COE (aa 499–638), SS2 (aa 748–755), SS6 (aa 764–771) and 2C10 (1368–1,374) (Kim et al., 2016; Lara-Romero et al., 2018; Tian et al., 2021). In this study, we found that the nine identified PEDV strains had multiple mutations in the neutralization epitope regions of SS2 and SS6 compared to CV777, which further validates that the currently prevalent PEDV strains are mutant PEDV, which may account for the poor protection of pigs against CV777-based vaccines.

PoRV is the second most common viral pathogen causing diarrhea in pigs. The 13 PoRV strains in this study were dominated by the G9 genotype, accounting for 61.54% of the total, followed by the G5 genotype, accounting for 15.38% of the total, which was consistent with the results of the previous study (Tao et al., 2023). Papp et al. collected and screened 77 original articles from various regions between 1976 and 2011 to investigate the distribution of porcine rotavirus group A genotypes. They found that G5 (45.8%) was by far the most common genotype among G typeable RVA strains, followed by G3 (11.2%) and G4 (9.6%) (Papp et al., 2013). 98 PoRV-positive samples were detected in 303 piglet diarrhea samples collected from 40 pig farms in 14 districts of Sichuan Province from 2017–2019, of which G9 was the dominant strain, accounting for 41%, while G4, G5, G26 and G3 accounted for 23 per cent, 28.2 per cent, 5.1 per cent and 2.7 per cent, respectively, (Zhou et al., 2021). In recent years, the detection rate of G9-type PoRV has been increasing, and it may be the latest epidemic G genotype PoRV in China. The prevalence of G9-type PoRV will undoubtedly increase the pressure of PoRV prevention and control, so it is necessary to further strengthen the isolation and detection of PoRV prevalent strains, and to screen for vaccine strains matching with the prevalent strains, with a view to providing a technological reserve for the control of PoRV epidemics (Hou et al., 2023).

## 5 Conclusion

To investigate the prevalence of major diarrhea-associated viruses in pigs, we examined 1,791 clinical samples from five provinces in southern China from 2021 to 2023. The results showed that PEDV was the most frequently detected virus, with prevalence rates ranging from 50.21 to 62.1%, PoRV was the second most prevalent virus detected in porcine diarrhea samples, with localized epidemics and increasing severity in the southern part of China, PDCoV had a tendency to become endemic, and TGEV and the newly emerged SADS-CoV remained sporadic. It is noteworthy that we reported for the first time the emergence of SADS-CoV in Jiangxi Province in southern China. The above pathogens were mainly mono-infections, and the dual mixed infections were dominated by PEDV+PoRV. Phylogenetic analyses showed that PEDV in South China over the past 3 years was mainly dominated by the GIIb variant of PEDV, and the PoRV detected was mainly dominated by G9.

## Data availability statement

The data presented in the study are deposited in the NCBI repository, accession number OR479072-OR479080 and OR475445-OR475457.

## Ethics statement

The Animal Ethics Committee of the Institute of Animal Husbandry and Veterinary, Jiangxi Academy of Agricultural Science for the studies involving animals because all samples were collected on commercial pig farms by pig veterinarians during routine diagnostic sampling after permission from the farm owner. No specific permits from an animal ethics committee were required. The studies were conducted in accordance with the local legislation and institutional requirements.

## Author contributions

FZ: Data curation, Formal analysis, Writing – original draft, Funding acquisition, Methodology. YL: Investigation, Resources, Funding acquisition, Writing – original draft. CL: Data curation, Formal analysis, Methodology, Writing – original draft. MT: Data curation, Investigation, Project administration, Writing – original draft. PW: Writing – original draft. BX: Writing – original draft. LX: Project administration, Writing – review & editing. HJ: Funding acquisition, Methodology, Writing – review & editing.
