# Supplementary material for: Epidemiological monitoring and genetic variation analysis of pathogens associated with porcine viral diarrhea in southern China from 2021 to 2023
Source: Front Microbiol. 2024 Mar 20;15:1303915. doi: 10.3389/fmicb.2024.1303915 (PMC10987963; doi:10.3389/fmicb.2024.1303915)
Supplement: Supplementary file 1 [file Data_Sheet_1.ZIP › supplementary material/Table S1-2.docx]

**Table S1** Statistics of premises positive for diarrhea-associated viruses

|  | PEDV | PDCoV | TGEV | PoRV | SADS-CoV |
| --- | --- | --- | --- | --- | --- |
| No.of premise tested | 213 | 213 | 213 | 213 | 213 |
| Positive premise | 162 | 82 | 8 | 155 | 11 |
| Positive rate | 76.06% | 38.50% | 3.76% | 72.77% | 5.16% |

**Table S2** Primers used in detection of PEDV, PDCoV, TGEV, PRoV, SADS-CoV and amplifying the S genes of PEDV and the VP7 genes of PoRV

| Primer name | Sequence (5'-3') | Target gene (Virus) | Product size |
| --- | --- | --- | --- |
| PEDV MF | GTATTGGTGGTGAGCGGAAT | ORF1 (PEDV) | 486 bp |
| PEDV MR | CCTGTTCCGCCATTCTATCA |  |  |
| PDCoV NF | CCAAACGCAACCCCAACAATCC | Nucleocapsid (PDCoV) | 329 bp |
| PDCoV NR | CTTCTCAGTGTCTGCAGAGCCG |  |  |
| TGEV SF | TATTTGTGGTTTTGGTTATAATGC | S gene (TGEV) | 870 bp |
| TGEV SR | GGCTGTTTGGTAACTAATTTGCCA |  |  |
| PoRV F | TATTCAAATATAAGTGATTTAATTCAAC | VP6 (PRoV) | 298 bp |
| PoRV R | TAATACCTGACAGCTTTCTTAATGC |  |  |
| SADS-CoV F | ACACCCAAACCAAGAAGCAG | Nucleocapsid (SADS-CoV) | 497 bp |
| SADS-CoV R | TCCACCATCTCAACCTCYTC |  |  |
| SA F | ATCTTCTGGCGTAATTCCACA | S gene (PEDV) | 1033 bp |
| SA R | CACCTTACCATGCACCAAAGT |  |  |
| SB F | ATGATTGGTCCCGTGTTGCGACA |  | 1032 bp |
| SB R | GCAATAAGGTTGGCACCACTA |  |  |
| SC F | GCCAACTCAAGTGTTCTCAGG |  | 1006 bp |
| SC R | AACAGGCGTGTTGTAAAGCTG |  |  |
| SD F | AGGGAGTTGCCTGGTTTCTTC |  | 1004 bp |
| SD R | AATGGCTTGGAAGTTGTGTTG |  |  |
| SF F | ACAGACGGATGTTCTACAGCG |  | 1041 bp |
| SF R | AATAATCAACCAAACCCACCA |  |  |
| SG F | GCAGATTTAGAGCAGCGTTCA |  | 1092 bp |
| SG R | GGTGACAAGTGAAGCACAGAT |  |  |
| VP7F | GGCTTTAAAAGAGAGAATTTCCGTCT | VP7 gene  (PoRV) | 1062 bp |
| VP7R | TCTAAATTCTGTAGTAAAAAGCAGCTG |  |  |
